# Lactate modulates zygotic genome activation through H3K18 lactylation rather than H3K27 acetylation

**DOI:** 10.1007/s00018-024-05349-2

**Published:** 2024-07-11

**Authors:** Yanhua Zhao, Meiting Zhang, Xingwei Huang, Jiqiang Liu, Yuchen Sun, Fan Zhang, Na Zhang, Lei Lei

**Affiliations:** 1 Department of Histology and Embryology, Harbin Medical University, Harbin, 150081 China; 2 State Key Laboratory of Female Fertility Promotion, Center for Reproductive Medicine, Department of Obstetrics and Gynecology, Peking University Third Hospital, Beijing, China; 3 Key Laboratory of Assisted Reproduction (Peking University), Ministry of Education, Beijing, China

**Keywords:** Lactate, Histone lactylation, Histone acetylation, Zygotic genome activation (ZGA), Embryo culture medium

## Abstract

In spite of its essential role in culture media, the precise influence of lactate on early mouse embryonic development remains elusive. Previous studies have implicated lactate accumulation in medium affecting histone acetylation. Recent research has underscored lactate-derived histone lactylation as a novel epigenetic modification in diverse cellular processes and diseases. Our investigation demonstrated that the absence of sodium lactate in the medium resulted in a pronounced 2-cell arrest at the late G2 phase in embryos. RNA-seq analysis revealed that the absence of sodium lactate significantly impaired the maternal-to-zygotic transition (MZT), particularly in zygotic gene activation (ZGA). Investigations were conducted employing Cut&Tag assays targeting the well-studied histone acetylation and lactylation sites, H3K18la and H3K27ac, respectively. The findings revealed a noticeable reduction in H3K18la modification under lactate deficiency, and this alteration showed a significant correlation with changes in gene expression. In contrast, H3K27ac exhibited minimal correlation. These results suggest that lactate may preferentially influence early embryonic development through H3K18la rather than H3K27ac modifications.

**Supplementary Information:**

The online version contains supplementary material available at 10.1007/s00018-024-05349-2.

## Introduction

An optimal culture medium system is essential for successful in vitro embryo culture and scientific research. Mouse embryo culture systems have been particularly valuable for studying human embryo culture, given the similarity in metabolic parameters between mouse and human [1–3]. Studies on mouse preimplantation embryo culture medium began in the 1950s [4, 5], and now there are well-established systems such as CZB [6] and KSOM [2, 7], which are widely used for mouse embryos culture in vitro. Various culture medium systems have been shown to support embryo development to the blastocyst stage [6–9], most contain a few basic components including glucose, pyruvate, lactate, and inorganic salts. Glucose supports the development of 8-cell embryos, but not 2-cell embryos [10, 11]. Pyruvate and lactate serve as energy sources in the medium and allow the development of 2-cell mouse embryos into blastocysts [10, 12]. Pyruvate plays a crucial role in oocyte maturation and the first cleavage division [13]. In a previous study, pyruvate was found to be essential for initiating zygotic genome activation (ZGA) and for the selective translocation of key mitochondrial TCA (tricarboxylic acid) cycle proteins to the nucleus, allowing for epigenetic remodeling and ZGA [14]. In some medium systems, like Krebs–Ringer bicarbonate with glucose and albumin, lactate must be added exogenously for successful embryo culture [15]. Although lactate is an integral component of embryo culture, little is known about its specific roles in embryonic development.

Excess accumulation of lactate has been shown to inhibit histone deacetylase activity and alter gene expression in human colon cancer cells (HCT116 cells) and macrophages cells respectively [16, 17]. Furthermore, in both normal kidney and Renal Cell Carcinoma (RCC) cells, lactate has been found to increase global H3 and H3K9ac by inhibiting SIRT1, a class of histone deacetylase [18]. More recently, histone lactylation has been discovered as a new epigenetic modification [19], with lactate potentially serving as a substrate for histone lysine lactylation and regulating various biological processes such as cancer invasion [20, 21], macrophage polarization [19, 22, 23], and ESCs cell fate determination [24]. Twenty-eight lactylation sites has been identified on core histones in human and mouse cells. During the process of macrophage polarization, changes in H3K18 lactylation (H3K18la) have been reported to exhibit a significant positive correlation with gene expression [19]. With this in mind, our study aimed to explore whether lactate in the embryo culture medium could potentially affect gene expression during embryonic development via histone lactylation or acetylation.

Our investigation compared the development of mouse embryos in mKSOM culture medium with or without lactate. We observed that lactate depletion led to developmental arrest at the late G2 phase of the 2-cell stage. Despite the decrease in components of the MPF complex at cell cycle checkpoints during the G2/M transition, the addition of exogenous agonist failed to rescue embryonic development. RNA-seq analysis revealed that the absence of sodium lactate affected the process of maternal-to-zygotic transition (MZT), particularly ZGA. Lactate deficiency led to a significant reduction in H3K18la, and the alteration of H3K18la was found to be responsible for ZGA. Contrary to expectations, lactate deficiency resulted in a partial upregulation of H3K27ac, which did not appear to be associated with defective gene expression. These results suggest that lactate regulates ZGA through H3K18la rather than H3K27ac.

## Materials and methods

### Antibodies

The following antibodies were used for immunostaining or western blotting: anti-H3S10ph (Abcam, #ab5176), anti-α-tubulin (Proteintech, #11224-1-AP), anti-γH2AX (Abcam, #ab2893), anti-CyclinB1 (Proteintech, #28603-1-AP), anti-CDK1 (Proteintech, #19532-1-AP), anti-SIRT1 (CST, #8469s), anti-H3K9ac (Sigma-Aldrich, #06-942), anti-H3K18ac (Sigma-Aldrich, #SAB5600233), anti-H3K27ac (CST, #8173s) and anti-H3K18la (PTM BIO, #1427RM). For secondary antibodies, we used goat anti-mouse secondary antibody, Alexa Fluor 488 (Invitrogen, #A-11001), goat anti-rabbit secondary antibody, Alexa Fluor 488 (Invitrogen, #A-11008), anti-rabbit HRP-linked antibody (CST, #7074s), and HRP-conjugated monoclonal mouse GAPDH (Kangchen, #KS-5G5).

### Mouse embryo culture

All experiments were performed in accordance with ARRIVE guidelines and Regulation. The study involving animal care and experiments was conducted in compliance with the guidelines set forth by the Animal Research Committee of Harbin Medical University. To obtain mouse embryos, 6-week-old ICR superovulated female mice were given a peritoneal injection of 7.5 IU of pregnant mare serum gonadotropin (PMSG), followed by 7.5 IU of human chorionic gonadotropin (hCG) 46–48 h after PMSG. The mice were then crossed with ICR males. At 18 h post hCG injection, the zygotes were isolated in M2 medium, washed in mKSOM medium, and subsequently transferred to the appropriate culture medium (+ L or − L) for further development. The mKSOM used in the study was a modified version of KSOM medium that contained the same salts, glucose, and pyruvate concentrations (95mM NaCl, 2.5mM KCl, 0.35mM KH_2_PO_4_, 0.20mM MgSO_4_, 25mM NaHCO_3_, 1.71mM CaCl_2_, 0.01 mM EDTA, 0.20mM glucose, 0.20mM pyruvate), but lacked all amino acids and BSA. The osmolarity of the medium was maintained using 0.1% poly vinyl alcohol (PVA).

### RNA extraction, reverse transcription and qRT-PCR

The RNA extraction was performed using the RNeasy Mini Kit (QIAGEN, #74134). To remove genomic DNA, the RNAse-Free DNase Set (QIAGEN, #79254) was employed. PrimeScript RT Master Mix (Takara, #RR036A) was used to obtain cDNA, and qRT-PCR was performed using TB Green Premix Ex Taq II (Takara, #RR82WR). Each sample was replicated three times. The primer sequences are provided in Table S1, and all steps were conducted following the handbooks.

### Immunofluorescence and confocal microscopy

The embryos were fixed in 4% paraformaldehyde (PFA) at room temperature (RT) for 30 min, followed by permeabilization with 0.1% Tween-20 at RT for 30 min. Subsequently, blocking was carried out with 1% BSA for 1 h at RT. The embryos were then incubated with the primary antibody overnight at 4 °C, washed three times with a washing solution, and incubated with the secondary antibody for 1 h at RT. The embryos were then stained with 5 µg/mL DAPI for 5 min and washed three times again. Finally, the embryos were mounted on glass slides and examined using a confocal laser-scanning microscope (Nikon, NIS Elements Analysis).

### Western blot

Total protein was extracted from 100 embryos per sample, separated by SDS PAGE and transferred to a PVDF membrane. The membrane was then cut into strips according to the marker sizes and incubated with the primary antibody overnight at 4 °C. After three washes, the membrane was incubated with an HRP-conjugated secondary antibody for 1 h at room temperature. The resulting image was examined using an enhanced chemiluminescence detection kit (Servicebio, #G2020-1, -2).

### EdU staining

The BeyoClick EdU-594 (Beyotime, #C0078S) was used to detect whether embryos grown in the − L group could replicate DNA in the S-phase. Embryos were cultured with or without lactate, and 100 μM of EdU was added to the cultures at 30 h post-hCG. The immunofluorescence staining was carried out at 48 h post-hCG, and the ratio of reaction buffer was determined based on the instructions. The signal was visualized using a confocal laser-scanning microscope.

### EU incorporation

The EU incorporation assay was performed using the Click-iT RNA Imaging Kit (Invitrogen, #C10330). At 46 h post hCG, two-cell stage embryos from either the + L or − L medium were transferred to a 1 mM EU culture medium, which was prepared by diluting the medium with or without lactate using mKSOM, and then cultured for 2 h. The subsequent procedures were performed following the kit's instructions. The fluorescence signal was quantified as the average intensity, and the Image J software was utilized for EU signal quantitation.

### Rescue experiment of exogenous drug addition

The exogenous added drugs involved in this paper are all directed to be added into − L culture medium. As reported in existing articles, the final concentration of OA added into − L group is 2.5μM. At 48 h post-hCG, the embryos are transferred from − L culture medium to the culture medium containing OA, which is cultured for 3h and then transferred into − L culture medium [26]. In addition to OA, other reagents such as lactate, sodium chloride, sodium acetate and lac-CoA were added according to the molar concentration of sodium lactate substance in + L group, and were immediately transferred to the corresponding culture medium after the zygotes were isolated.

### Protein synthesis assay

To detect protein synthesis, we used the Click-iT HPG Alexa Fluor Protein Synthesis Assay Kits (Life Technologies, #10428). Component A was diluted with mKSOM medium with or without lactate at a 1:1000 ratio. Two-cell stage embryos from + L or − L groups were transferred to the diluted medium and incubated at 37 °C with 5% CO_2_ for 2 h. After washing with PBS, subsequent operations were performed following the kit's instructions.

### RNA sequencing

We collected zygotes and two-cell embryos at 18 and 52 h post-hCG, respectively. Each sample was replicated three times. Low input RNA-seq analysis was conducted by Frasergen Company. Quantitative and statistical analyses were performed accordingly.

### RNA-Seq data analysis

The RNA sequencing raw data underwent preprocessing using Trimmomatic (v0.39) to eliminate sequences containing adaptors. Subsequently, the cleaned data were aligned to the GRCm39 reference genome using hisat2 (v2.2.1), and gene expression levels were quantified through the feature Counts software in subread (v2.0.6). Differential expression analysis utilized the raw counts generated by feature Counts, and the R package edgeR was employed for statistical analysis. Genes were deemed significantly differentially expressed based on a p-value cutoff of 0.05 and a fold change cutoff of 2.

### CUT&Tag data analysis

For the raw data derived from CUT&Tag sequencing, adaptor removal was performed using Trimmomatic (v0.39), and reads exceeding 36 base pairs in length were retained. The resulting clean data were aligned to the GRCm39 reference genome using bowtie2 (v2.5.1). PCR replicates were eliminated through sambamba markdup removal. Subsequent analyses involved normalizing read counts by calculating reads per kilobase per million mapped reads (RPKM) for 100 base pair genomic bins. To mitigate batch and cell type variations, RPKM values for the entire genome underwent additional normalization through Z-score transformation. For visualization of the CUT&RUN signal in the UCSC Genome Browser, RPKM values were generated based on 100 base pair windows. Peaks were identified using macs2 (v2.2.9.1) with parameters: -broad -nolambda -nomodel. The genomic distribution of peaks was plotted using deeptools (v3.5.4). Differential analysis of CUT&Tag data employed the R package DiffBind to identify significantly differentially expressed peaks, utilizing a p-value cutoff of 0.05 and a fold change cutoff of 2.

### Defining ZGA gene and maternal decay gene

For ZGA genes, those exhibiting a fold change cutoff of 3 and a p-value cutoff of 0.05 in the comparison between the two-cell stage and the zygote were designated as ZGA genes. Similarly, genes with a fold change cutoff of 1/3 and a p-value cutoff of 0.05 in the two-cell stage compared to the zygote were categorized as maternal decay genes. To analyze Fig. 5I, we first selected a list of ZGA genes. We then conducted a differential expression analysis of these genes using RNA-Seq data from the + L and − L groups. Based on this analysis, we categorized the ZGA genes into three groups: ZGA_down, ZGA_normal, and ZGA_up. Subsequently, we performed differential analysis on these three groups using the H3K18la CUT&Tag data from the + L and − L groups. This analysis was carried out with the DiffBind package. Based on the differential analysis results, we further classified the genes into three categories: strong, median, and weak.

### Statistical analysis

All experiments were conducted a minimum of three times, unless otherwise indicated. The statistical analysis was performed using the student *t* test to determine the significance of differences between groups, and a p-value of less than 0.05 was considered statistically significant. All values are reported as the mean ± SD.

## Results

### The indispensable of lactate in the early stage of embryonic development

**Fig. 1:**
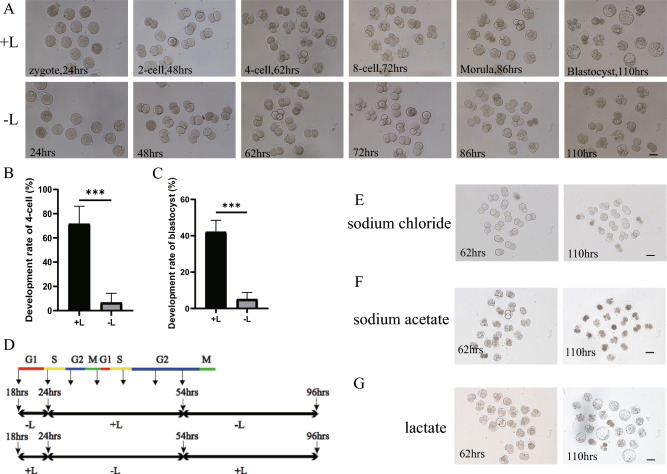
Lactate is important for early embryonic development in mice. **A** Embryonic development of embryos in the + L and − L groups at different time points. Scale bar: 50 µm. **B** 4-cell formation rate of embryos in the + L and − L groups. **C** Blastocyst formation rate of embryos in the + L and − L groups. **D** Cell-cycle phases in 1- and 2-cell embryos (Aoki et al., 1997). Schematic diagram of embryo transition in medium with and without sodium lactate at different time points. **E** Embryos cultured in − L medium that was added with sodium chloride at different time points. Scale bar: 50 µm. **F** Embryos cultured in − L medium that was added with sodium acetate at different time points. Scale bar: 50 µm. **G** Embryos cultured in − L medium that was added with lactate at different time points. Scale bar: 50 µm

To ensure that amino acids and proteins would not compensate for the lack of lactate via alternative metabolic pathways, we employed modified KSOM (mKSOM) for embryo culture with only sodium lactate, glucose, pyruvate, salts/buffer, and polyvinyl alcohol [14]. Zygotes were isolated at 18 h post-human chorionic gonadotropin (hCG) injection and cultured in + L or − L medium, which with or without sodium lactate. Embryos grown in + L medium showed 71.6% of embryos cleavage to the four-cell stage at 48 h post hCG, and 42.1% of embryos developed to blastocysts by 110 h. Conversely, most embryos nourished with − L medium were arrested at the 2-cell stage, with only 6.7% reaching the 4-cell stage, and merely 5.1% transitioning to the blastocyst stage (Fig. 1A–C, Table 1). By switching the medium between + L and − L at various time points, when embryonic development occurred outside the designated window, we observed no discernible differences between − L/ + L/− L group and the + L group, suggesting that the critical period for the lactate requirement was mapped to a 30 h time window between 24 and 54 h post hCG (Fig. 1D and Table S1). To exclude the possibility that the observed phenotypes were due to differences in osmotic pressure and pH between + L and − L medium, developmental rescue experiments were designed with different components. The addition of sodium chloride to − L group to maintain consistent osmotic pressure as in + L group had no positive effect on development (Fig. 1E). Furthermore, when sodium acetate was added to compensate for osmotic pressure and pH, the embryos still failed to pass the 2-cell stage (Fig. 1F). However, when lactate replaced sodium lactate in the − L group, even though the medium was acidic, the embryo development rate reached similar level as those grown in + L group (Fig. 1G). These results suggest that the arrest of embryos grown in − L group is due to lactate ions deficiency, rather than other differences between + L and − L medium.

**Table 1:** Developmental phenotype of embryos cultured in + L and − L medium

	Experiments	Embryos	4-cell	Blastocyst
	(n)	(n)	(%)	(%)
+ L	4	108	71.6 ± 14.5	42.1 ± 6.4
− L	4	110	6.7 ± 7.6***	5.1 ± 3.7***

Four experimental replicates were performed. 4-cell, statistics were performed 62 h post hCG injection. Blastocysts were counted 110 h post hCG injection. Two-tailed Student’s t-test was used for the statistical analysis. ***P < 0.001

### Lactate depletion arrests embryonic development at late G2 phase of the 2-cell stage

**Fig. 2:**
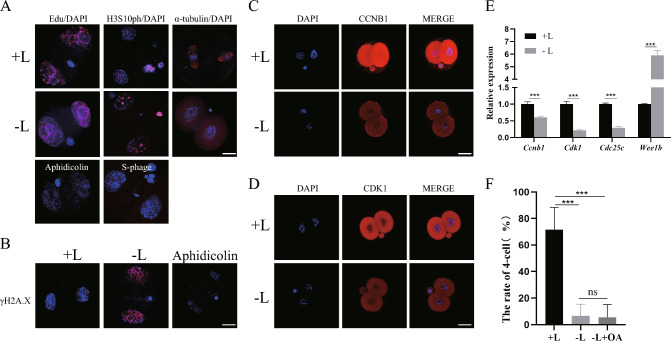
Depletion of lactate in the culture medium leads to developmental arrest at the G2-M transition stage of the 2-cell stage and a reduction in the MPF complex. **A** Immunostaining shows the embryos EdU incorporation marking S phase, Aphidicolin as the positive control, H3S10ph staining marks G2 phase, S-phage as the negative control, α-tubulin staining marks M phase. Scale bar: 20μm. **B** Embryos culture in + L or − L display similar γH2A.X staining (red), with Aphidicolin as the positive control. Scale bar: 20 μm. **C** Images of embryos immunostained with antibodies against CCNB1 between + L and − L group. Scale bar: 20μm. **D** Images of embryos immunostained with antibodies against CDK1 between + L and − L group. Scale bar: 20 μm. **E** qPCR of genes involved in MPF transcripts for + L and − L group embryos, ***P < 0.001. **F** Statistics of 4-cell rate of + L, − L group and rescue group with exogenous OA in − L, ***P < 0.001. ns indicates no statistical difference

To identify the specific cell cycle phase of developmental arrest resulting from lactate depletion, we added EdU to embryos at 30 h post hCG and imaged them at 48 h post hCG to assess whether S phase DNA replication occurred in embryos of − L group. We used immunofluorescence staining (IF) to detect histone H3 serine 10 phosphorylation (H3S10ph) at 48 h post hCG to visualize whether the embryo entered G2 phase, and also labeled M phase with α-tubulin at 52 h post hCG. The results showed that embryos grown in − L medium progressed through S phase and entered G2 phase similarly to those grown in + L medium (Fig. 2A). However, at 52 h post hCG, we observed that almost all the chromatin of embryos in + L group had condensed into chromosomes, and part of the embryos had the metaphase spindle structure, while there were almost no agglutinated chromosomes and no embryos in the − L group had the metaphase phase spindle structure (Fig. 2A). Therefore, embryos grown in − L medium arrest specifically at the G2-M transition phase of the 2-cell stage. To explore whether the 2-cell block was due to the stress caused by the absence of lactate leading to DNA damage, we used γH2AX, a marker of DNA damage in eukaryotes, to demonstrate DNA damage. We observed minimal γH2AX signals in 2-cell embryos from both + L and − L groups, while embryos treated with aphidicolin as a positive control showed strong γH2AX signals (Fig. 2B). These results indicated that depletion of lactate in the culture medium leads to developmental arrest at late G2 phase of the 2-cell stage, which is not due to DNA damage.

The G2 phase is the second gap in the cell cycle during which the cell prepares for mitosis. The G2-M transition is regulated by the MPF complex, consisting of CDK1 and CCNB1. CDK1 is a serine/threonine protein kinase that can be phosphorylated and dephosphorylated at Y15 and T14 by WEE1 and CDC25c, respectively. CCNB1 is a cyclically expressed protein required for CDK1 activation [25]. We investigated whether there were differences in MPF complex expression between the + L and − L group. We found that the RNA levels of *Cdk1*, *Ccnb1*, and the positive regulator *Cdc25c* were downregulated, while the negative regulator *Wee1b* was upregulated in the − L group (Fig. 2E). In addition, both the protein levels of CDK1 and CCNB1 decreased as shown by IF. Moreover, CCNB1was found to be present in both the nucleus and cytoplasm of late 2-cell embryos in the + L group, while it was solely located in the cytoplasm in the − L group, consistent with prior research (Fig. 2C, D) [26]. To explore whether the disparity in MPF expression was the cause for the developmental arrest of embryos in the − L group at the late 2-cell stage, we conducted a developmental rescue experiment by supplementing − L medium with okadaic acid (OA), a CDK1 agonist. However, the addition of OA could not effectively rescue the 2-cell block (Fig. 2F). These results suggest that the reduction of MPF from lactate depleting is not the most important reason for 2-cell arrest.

### Lactate is crucial for the MZT

**Fig. 3:**
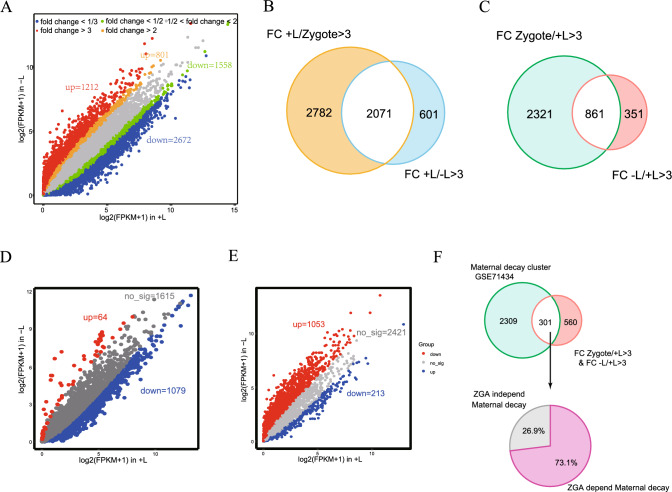
Lactate is crucial for the MZT. **A** The scatter plots show the different genes between the + L and − L groups. **B** The Venn diagrams illustrate the intersection of transcripts significantly upregulated during the MZT (fold change [+ L/zygote] > 3) in + L embryos and transcripts that are downregulated in response to lactate deficiency (fold change [+ L/− L] in late 2-cell > 3). **C** The Venn diagrams illustrate the intersection of transcripts significantly downregulated during the MZT (fold change [zygote/ + L] > 3) in + L embryos and transcripts that accumulate in response to lactate deficiency (fold change [− L/ + L] in late 2-cell > 3). **D** Scatter plots depicting the changes in expression levels of late 2-cell ZGA genes in response to lactate deficiency. **E** Scatter plots illustrating the changes in expression levels of maternal decay genes under conditions of lactate deficiency. **F** The top Venn diagrams depict the overlap in transcripts between the GSE71434-published maternal decay cluster and maternal decay transcripts that are not degraded in response to lactate deficiency (fold change [zygote/ + L] > 3 & fold change [− L/ + L] in late 2-cell > 3). The pie chart below illustrates the proportion of overlapping genes categorized as ZGA-dependent and ZGA-independent maternal decay genes.

In early embryonic development across all animals, the "maternal-to-zygotic transition (MZT)" marks a crucial step, during which developmental control shifts from the maternal genome to the zygotic genome. This event involves the elimination of the majority of maternal RNAs and proteins, activation of transcription in the zygotic genome [27]. As lactate is vital for development between 24 and 54 h post hCG, precisely coinciding with the key MZT period, we conducted global RNA-seq analyses on late 2-cell embryos in both + L and − L groups, as well as derived zygotic embryos. Gene expression, measured as fragments per kilobase of transcript per million mapped reads (FPKM), exhibited high correlation in duplicate samples (Figure S1A). Our analysis identified 6249 differential genes (DEG) (FDR < 0.05, fold change > 2) when comparing + L medium grown embryos with those grown in − L medium. In the lactate-depleted group, 4236 genes were downregulated, and 2013 genes were upregulated. Even with a stricter DEG definition (FDR < 0.05, fold change > 3), the down-regulated genes in the − L group at the late 2-cell stage were more than twice those upregulated compared to the + L group (2672 versus 1212) (Fig. 3A). We defined genes with a significant > three fold increase in mRNA levels in late 2-cell embryos of the + L group compared to the zygote as ZGA genes, and those with > three fold decreases as maternal degradation genes [28]. Of the 2672 down-regulated transcripts, 2071 were ZGA genes (Fig. 3B), while 861 of the 1212 up-regulated genes were maternal degradation genes (Fig. 3C). Both ZGA and maternal degradation patterns were significantly disturbed in the − L group (Fig. S1B, S1C). Further verification using published ZGA and maternal gene lists confirmed that the down-regulated ZGA genes in the − L group approximately 60% belong to the ZGA genes category (1272/2071), and more than half of the up-regulated maternal degradation genes belonged to the maternal gene list (451/861) (Figure S1D, S1E). Comparing with the ZGA and maternal degradation gene list by Zhang Yi et al. [29], we observed more down-regulated genes in the − L group for ZGA genes (Fig. 3D), while for maternal degraded genes, more genes were up-regulated in the − L group (Fig. 3E). Through these comparisons, we found the deficiency of sodium lactate significantly disrupted both ZGA and maternal degradation processes. Maternal degradation abnormalities and ZGA failure mutually influenced each other, potentially leading to 2-cell arrest. Given the substantial impact of lactate deficiency on both processes, we further classified shared genes between abnormal maternal degradation genes in the − L group and previously reported maternal degradation genes into ZGA-dependent and ZGA-independent categories. Results indicated that 73.1% of common genes were ZGA-dependent maternal degradation genes, a ratio significantly higher than that of ZGA-independent maternal degradation genes (Fig. 3F), suggesting that ZGA failure in the − L group may be responsible for the incomplete degradation of numerous maternal genes.

### The depletion of lactate leads to ZGA failure

**Fig. 4:**
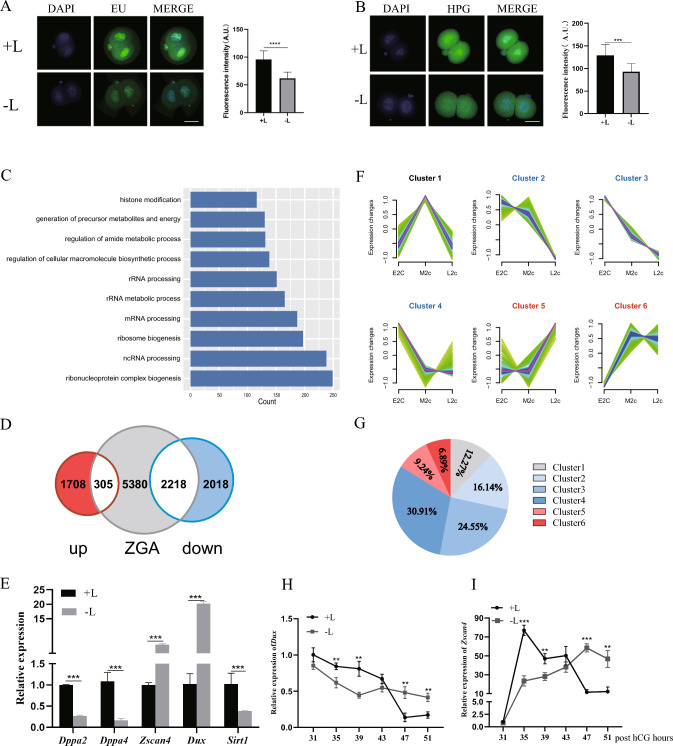
The depletion of lactate leads to the failure of ZGA. **A** Left, embryonic EU incorporation assayed in + L or − L medium (green), DAPI counterstain (blue). Scale bar: 20 μm. Right, − L embryos show a significant reduction of EU incorporation. ****P < 0.0001. **B** Left, Embryonic HPG incorporation assayed in + L or − L medium(green), DAPI counterstain (blue). Scale bar: 20 μm. Right, − L embryos show a significant reduction of HPG incorporation. ***P < 0.001. **C** Gene Ontology (GO) enrichment for the down-regulated genes in − L group, (FDR < 0.05, and fold change > 2). **D** Venn diagrams shows the overlap between ZGA genes and up-regulated, down-regulated genes in − L group (FDR < 0.05, and fold change > 2). **E** qPCR analysis of ZGA gene transcripts in + L and − L embryos. ***P < 0.001. **F** Fuzzy c-means clustering identifies six distinct temporal patterns of differential gene expression. The *x*-axis represents three 2-cell developmental stages, while the *y*-axis represents log2-transformed, normalized intensity ratios at each stage. **G** The pie chart shows the distribution of genes in each cluster mentioned in **E**. **H**. qPCR analysis of *Dux* in the + L and − L groups at 31 h, 35 h, 39 h, 43 h, 47 h, and 51 h post hCG.** P < 0.01. I. qPCR analysis of *Zscan4* in the + L and − L groups at 31 h, 35 h, 39 h, 43 h, 47 h, and 51 h post hCG. **P < 0.01, ***P < 0.001

To evaluate the newly synthesized RNA in lactate-depleted 2-cell embryos, we used EU staining for total de novo transcripts and L-HPG to detect the nascent protein synthesis, and found a significant downregulation of RNA and protein syntheses in the lactate-depleted group (Fig. 4A, B). To further explore the effects of lactate deficiency on ZGA, we compare DEG in the two groups (FDR < 0.05, fold change > 2). The functions of the down-regulated DEG in − L group were enriched in ribosome biogenesis and RNA processing, etc. (Fig. 4C), indicating aberrant ZGA. We compared the DEG with the ZGA gene list of Ken-ichiro Abe et al*.* [30], and found that 2218 of the 4236 downregulated genes belong to the ZGA genes category, whereas 305 of the 2013 upregulated genes are the ZGA genes (Fig. 4D). Some of the DEG were verified by qPCR, and the results were consistent with those of RNA-seq analysis (Fig. 4E). Interestingly, ZGA related genes such as *Dppa2*, *Dppa4*, and *Sirt1* were downregulated in the lactate depletion group. However, the representative ZGA genes, *Zscan4* and *Dux*, were highly expressed in the lactate-depleted group, as observed in qPCR results (Fig. 4E). These led us to hypothesize that these ZGA related genes were likely activated normally but not undergoing the expected decline in late 2-cell stage. To test our hypothesis, we further divided the 2-cell stage into early 2-cell (E2c, 31-32h after hCG), middle 2-cell (M2c, 39-40h after hCG) and late 2-cell (L2c, 46-48h after hCG), and analyzed 1320 up-regulated DEGs, subsequently performed soft clustering on the genes based on their expression trends reference single cell sequencing data [31]. We categorized them into six clusters in total, with Cluster 1 showing an increase followed by a decrease in gene expression during development from E2c to L2c, while Clusters 2, 3, and 4 showing a gradual decrease in gene expression. Clusters 5 and 6 showed the opposite trend. As expected, most of the up-regulated genes belonged to the first four clusters, with only 16.13% genes falling into cluster 5 and cluster 6 (Fig. 4F, 4G). Additionally, we also collected 2-cell embryos from the + L group and − L group at 31 h, 35 h, 39 h, 43 h, 47 h, and 51 h post hCG for qPCR verification of *Dux* and *Zscan4* expression. We found that the transcription levels of *Dux* and *Zscan4* were higher in the + L group than that in the − L group before 43 h post hCG. However, slower transcript decay in embryos grown in − L medium, *Dux* and *Zscan4* expression levels were higher in the − L group than that in the + L group after 47 h post hCG (Fig. 4H, I). The result is consistent with our hypothesis that genes belonged to ZGA up-regulated in the − L group are more likely to have not completed effective degradation during the late 2-cell stage. In conclusion, our findings suggest that depletion of lactate leads to the failure of ZGA.

### The alteration of H3K18la is responsible for the ZGA failure

**Fig. 5:**
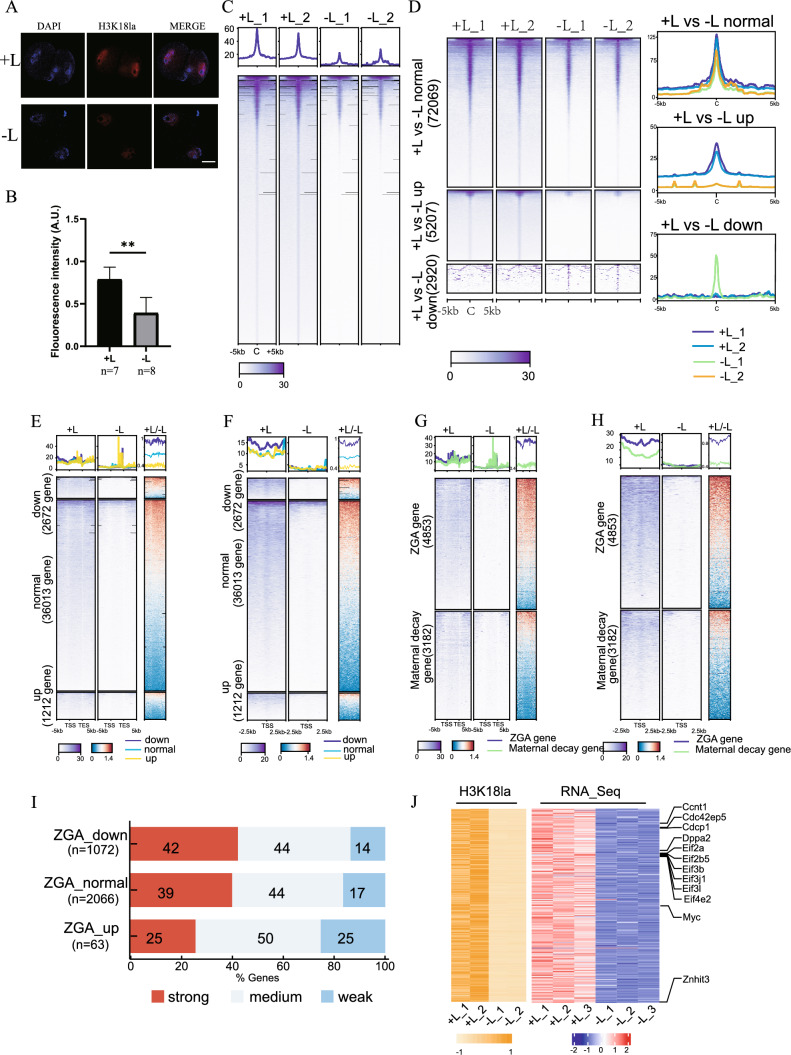
The histone lactylation is responsible for the ZGA failure. **A** Images of embryos immunostained with antibodies against H3K18la in the + L and − L groups. Scale bar: 20 μm. **B** Bar plot showing the H3K18la fluorescent intensity of 2-cell embryos derived from the + L and − L group. ** P < 0.01. **C** Heatmap shows H3K18la signals in late 2-cell embryos from the two groups, with and without sodium lactate. **D** All H3K18la domains from both + L and − L group were categorized into three clusters based on their differences. The C-domain represents the center. **E** The heatmap illustrates the enrichment of H3K18la signals in the gene bodies of DEGs between the + L and − L groups. The terms "down," "normal," and "up" refer to genes that are down-regulated, exhibit no significant difference, and are up-regulated, respectively, in the − L compared to + L. Average plots depict the enrichment of H3K18la signals in the + L compared with − L. **F** The heatmap showcases the enrichment of H3K18la signals in the promoters of DEGs between the + L and − L groups. Similar to (**C**), "down," "normal," and "up" indicate genes that are down-regulated, show no significant difference, and are up-regulated, respectively, in the − L compared to + L. Average plots display the enrichment of H3K18la signals in the + L compared with − L group. **G** The heatmap illustrates the enrichment of H3K18la signals in the gene bodies of maternal decay genes (fold change [zygote/ + L] > 3) and ZGA genes (fold change [+ L/zygote] > 3) between the + L and − L groups. Average plots demonstrate the enrichment of H3K18la signals in the + L compared with − L group. **H**. The heatmap depicts the enrichment of H3K18la signals in the promoters of maternal decay genes (fold change [zygote/ + L] > 3) and ZGA genes (fold change [+ L/zygote] > 3) between the + L and − L groups. Average plots show the enrichment of H3K18la signals in the + L compared with − L group. **I** The bar chart categorizes ZGA genes based on their expression changes under deficient lactate conditions and the H3K18la CUT & Tag signal measured in their gene body regions. **J** The heatmap demonstrates that many genes critical for ZGA are significantly down-regulated in the − L group, and the signal of H3K18la is also notably absent or down-regulated

Given lactate is known capacity to stimulate histone lactylation, we hypothesized that the absence of lactate in the embryo culture medium would result in a reduction of histone lactylation levels. We first performed IF on well-studied H3K18la site and found that the fluorescence intensity in embryos from the − L group was significantly lower than that in embryos from the + L group (Fig. 5A, B). Subsequently, we conducted the ultra-low-input CUT&Tag method with as few as 200 late 2-cell mouse embryos on this histone modification site between the + L and − L groups. Two replicates exhibited a high correlation (Fig S2A, S2B). The domain size distribution was notably concentrated on the promoter region and intergenic region (Fig S2C). Moreover, genes expressed at higher levels displayed greater enrichment of H3K18la signals compared to genes expressed at lower levels (Figure S2D), suggesting that H3K18la may function as an activating histone modification, consistent with prior research on embryonic stem cells (ESCs) [24]. A significant genome-wide downregulation of H3K18la was observed in the − L group (Fig. 5C). We further categorized the differences in H3K18la enrichment between the two groups into three clusters: down regulated, normal and up regulated cluster, revealing that the peaks with differences between the two groups were more down-regulated in the − L group (Fig. 5D). Correspondingly, these peaks were associated with more down-regulated genes (Fig S2E). Gene Ontology (GO) enrichment analysis indicated that the down-regulated genes were enriched for processes such as ribosome biogenesis and histone modification (Fig S2F). Subsequently, we classified RNA-seq data based on DEGs and explored the relationship between DEGs and differential peaks of H3K18la CUT&Tag data. Notably, the intensity changes of H3K18la peaks in down-regulated differential genes in the − L group were most pronounced, both in the gene body (Fig. 5E) and the promoter region (Fig. 5F). Regardless of whether considering our defined ZGA and maternal degradation genes or those gene lists defined by Zhang Yi et al. [29], the enrichment of H3K18la in the gene body (Fig. 5G, Fig S2G) or promoter (Fig. 5H, Fig S2H) of ZGA genes in the − L group showed a marked reduction. These results imply that the absence of sodium lactate leads to the downregulation of H3K18la modification, and a clear positive correlation exists between the change in H3K18la and gene expression, especially ZGA genes. Further examination of the correlation between ZGA gene expression changes in embryos subjected to lactate deficiency and H3K18la occupancy revealed that genes with strong H3K18la occupancy were significantly less expressed upon lactate deficiency compared to genes with weak occupancy (Fig. 5I). Many of the ZGA genes with significantly down-regulated H3K18la modifications were lowly expressed in the RNA-Seq data for the − L group. Notably, this subset includes several critical ZGA genes such as *Ccnt1* and *Dppa2*, as illustrated in Fig. 5J and Fig S2I. These findings suggest that the alteration of H3K18la is accountable for the ZGA failure.

### H3K27ac is not responsible for ZGA failure due to lactate deficiency

**Fig. 6:**
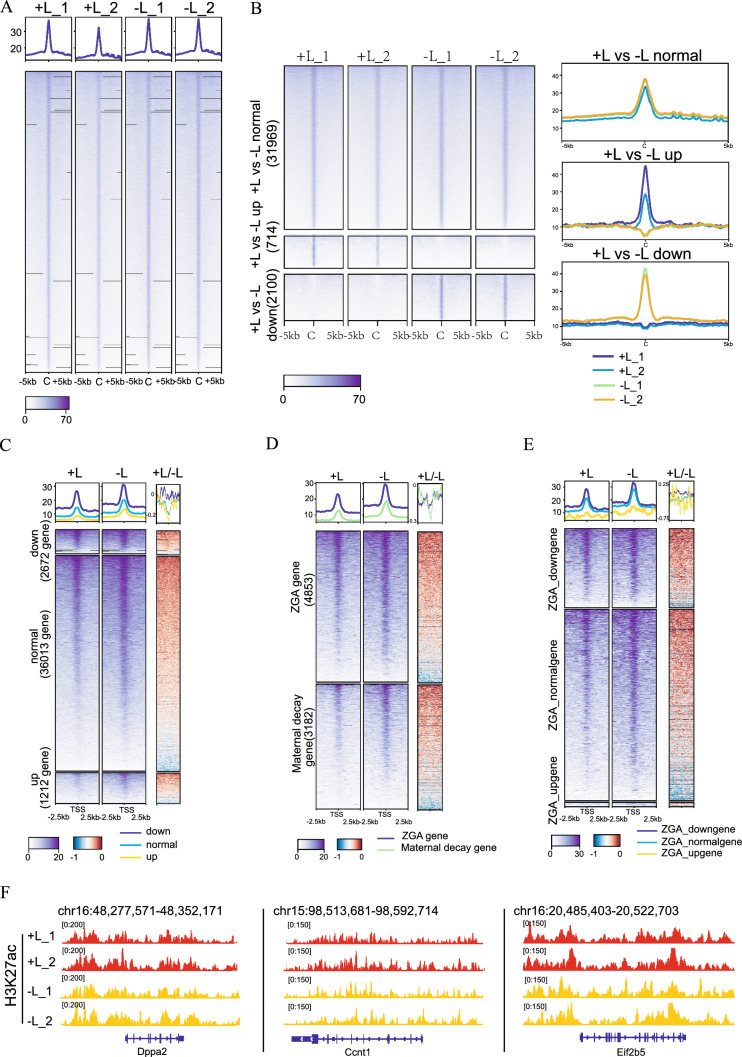
H3K27ac is not responsible for ZGA failure due to lactate deficiency. **A** The heatmap displays H3K27ac signals in late 2-cell embryos from two groups, with and without sodium lactate. **B** All H3K27ac domains from both + L and − L groups were categorized into three clusters based on their differences. The C-domain represents the center. **C** The heatmap illustrates the enrichment of H3K27ac signals in the promoters of DEGs between the + L and − L groups. The terms "down," "normal," and "up" refer to genes that are down-regulated, exhibit no significant difference, and are up-regulated, respectively, in the − L compared to + L. Average plots depict the enrichment of H3K27ac signals in the + L compared with − L group. **D** The heatmap illustrates the enrichment of H3K27ac signals in the promoters of DEGs between the + L and − L groups. Average plots depict the enrichment of H3K27ac signals in the + L compared with − L group. **E** The heatmap illustrates the enrichment of H3K27ac signals in the promoters of DEGs that belong to ZGA genes (fold change [+ L/zygote] > 3) between the + L and − L groups. Average plots show the enrichment of H3K27ac signals in the + L compared with − L group. **F** Representative IGV snapshots show the enrichment of H3K27ac signal in both + L and − L groups

To investigate the effect of lactate on histone acetylation in mouse embryos, we first performed IF on typical histone acetylation modification sites, H3K9ac, H3K18ac, and H3K27ac, in 2-cell embryos from both the + L and − L groups. The staining results showed no significant differences in fluorescence intensity between the two groups (Figure S3A-S3F). To further confirm this result, we utilized the ultra-low-input CUT&Tag method to profile the genome-wide distribution of H3K27ac signals. H3K27ac has been reported to exhibit dynamic changes during early embryonic development reprogramming [29], Our findings demonstrated a robust correlation between our + L group data and public available late 2-cell embryo data obtained through a different profiling method and antibody (Figure S4A). Replicate samples exhibited similar peaks in H3K27ac levels (Figure S4B, S4C). H3K27ac occupied both genic and intergenic regions (Figure S4D), consistent with published research [29, 32]. However, in our study, there were no significant changes in genome-wide H3K27ac levels between the two groups (Fig. 6A). Upon further categorization of H3K27ac peaks into three clusters in the two groups, in contrast to H3K18la results, there were more up-regulated peaks and associated genes in the − L group (Fig. 6B, S4E). This outcome may be attributed to the decreasing trend of H3K27ac modification during zygote to 2-cell development (Figure S4F). In the − L group, due to the embryo development block, some H3K27ac modification areas that should have decreased were not reduced timely. Subsequently, we examined the differences in peaks corresponding to DEGs from RNA-seq data between the two groups, mirroring the approach used for H3K18la. Whether considering all DEGs in the two groups (Fig. 6C, S4G), or categorizing them based on maternal degradation or ZGA genes (Fig. 6D, S4H), or focusing solely on DEGs in ZGA (Fig. 6E, F, S4I), effective separation of H3K27ac peaks on the promoter and gene body was not achieved within these classes. These results suggest that the absence of lactate, resulting in changes in H3K27ac, may not be the causative factor for alterations in gene expression.

## Discussion

In our investigation, we observed a marked influence on embryo development, resulting in a 2-cell arrest, in the absence of sodium lactate in mKSOM medium [14] (devoid of all amino acids and BSA). A recent study by Jingyu Li et al. [33] indicated that the sole absence of lactate in the medium does not induce a 2-cell arrest, Upon comparing with our medium, it was evident that the medium employed by Jingyu Li et al. retained L-glutamine, a non-essential amino acid. We also investigated the removal of only sodium lactate from the KSOM medium, and no 2-cell arrest occurred. These findings suggest that amino acids and BSA may potentially compensate for the absence of sodium lactate through distinct metabolic pathways during early embryonic development. Notably, the deficiency of lactate has long been associated with the induction of embryonic development 2-cell arrest, dating back to the mid-twentieth century. This was demonstrated in seminal studies such as the 1957 Krebs–Ringer medium experiment, wherein the absence of lactate led to the arrest of embryonic development at the 2-cell stage [15]. While varying culture conditions may yield diverse embryonic development phenotypes, collectively, these studies underscore the pivotal role of lactate in early embryonic development that cannot be overlooked.

Further confirmation of the arrested cell cycle in our study showed that the lack of lactate resulted in embryo arrest in the late G2 phase, accompanied by a downregulation of essential components of the MPF complex. Although exogenous agonist of MPF complex components was added at cell cycle checkpoints during the G2/M transition, it failed to rescue embryonic development. Recent reports suggest that lactate may regulate the cell cycle by remodeling the anaphase promoting complex (APC/C) through binding and inhibiting the SUMO protease SENP1. The SUMOylation of APC4 directly affects the assembly of APC/C [34]. It is possible that lactate has a regulatory effect on other cyclin complexes, the regulation may not only through changes in protein expression levels but also through modifications in enzyme activity or the binding interactions between complex subunits. However, due to the limited number of embryos, this part of the study will be validated in cell lines in the future.

MZT is a crucial process in early embryonic development, which involves the elimination of the majority of maternal RNAs and proteins, activating transcription in the zygotic genome. Abnormalities in either maternal degradation or zygotic gene activation can result in a 2-cell arrest in embryos [28, 35–37]. Comparative RNA-seq analysis of embryos under lactate-deficient conditions revealed noticeable impacts on both processes. To determine whether abnormal maternal degradation or failed ZGA is more likely to be the cause of the 2-cell arrest, we utilized previously published dataset [38]. By comparing RNA-seq data before and after α-Amanitin treatment, we identified genes associated with ZGA-dependent and ZGA-independent maternal degradation. A significant proportion of genes exhibiting abnormalities in maternal degradation were found to be ZGA-dependent maternal degradation genes. This result suggests that the failure of ZGA is more likely to be the reason for the ineffective degradation of maternal degradation genes. We have substantiated the deficiency of lactate as a cause for the failure of ZGA through multiple approaches. Our findings are consistent with prior research, which illustrated that during the 2-cell stage, mouse embryos display notable metabolic activity characterized by substantial lactate production [32]. Recent studies by Jingyu Li and colleagues have also indicated that the absence of lactate leads to a failure in major ZGA [33], since we did not conduct RNA-seq on early 2-cell embryos in both groups, we have not been able to distinguish whether the impact on ZGA is related to major ZGA or minor ZGA.

We performed Cut&Tag targeting two relatively well-studied histone modification sites, H3K18la and H3K27ac. We observed a significant reduction in H3K18la modification in the absence of sodium lactate, and the changes in H3K18la modification showed a clear correlation with alterations in gene expression. This suggests that lactate may provide substrate support for H3K18la lactylation modification and the change of H3K18la lactylation modification further affects the gene expression. Unexpectedly, the impact on H3K27ac modification was minimal, and even more sites with increased H3K27ac were observed in the lactate-deficient group. This could be attributed to the embryo arrest, leading to ineffective erasure of H3K27ac during the zygotic stage. The differences in H3K27ac between the two groups showed almost no correlation with the change of gene expression. Despite H3K18la and H3K27ac modifications being considered as activating epigenetic marks, our analysis revealed distinct differences between them under the lactate-deficiency condition. Lactate is more likely to serve as a substrate for lactylation rather than acetylation, altering histone lactylation rather than acetylation. However, to comprehensively understand the impact of lactate on histone lactylation or acetylation modifications, analyzing only these two selected sites is not sufficient, further exploration of modifications at more sites is warranted.

Lactate is an essential energy source in cell culture and early embryonic development. Although the detection methods are limited, the significant down-regulation of ATP (Figure S5A) and ROS levels (Figure S5B) in lactate-deficient cultured embryos suggested that lactate plays a crucial role as an energy supplier in this process. Our RNA-seq data reveals that genes related to ribosome biogenesis were significantly down-regulated under lactate deficiency. Previous studies have brought to light the discovery of a protein complex known as eNoSC (energy-dependent nucleolar silencing complex), the complex establishes a connection between cellular energy levels and the synthesis of ribosomal RNA (rRNA) [39]. This suggests that, in the early stages of mouse embryonic development, lactate not only influences embryonic development through changes in epigenetic modifications but also plays an essential role as a classical energy supply, highlighting the need for further exploration and investigation.

### Supplementary Information

Below is the link to the electronic supplementary material.

Supplementary file1 (DOCX 1073 KB)

## Data Availability

All relevant data are within the paper and its Supporting Information files. The raw sequence reads have been deposited in NCBI National Library of Medicine at https://dataview.ncbi.nlm.nih.gov/object/PRJNA1043759?reviewer=7b6hq13153kqa99o4o0idub3fs under accession number PRJNA1043759.
