# Supplementary material for: Lactate modulates zygotic genome activation through H3K18 lactylation rather than H3K27 acetylation
Source: Cell Mol Life Sci. 2024 Jul 11;81(1):298. doi: 10.1007/s00018-024-05349-2 (PMC11335220; doi:10.1007/s00018-024-05349-2)
Supplement: Supplementary file 1 — Supplementary file1 (DOCX 1073 KB) [file 18_2024_5349_MOESM1_ESM.docx]

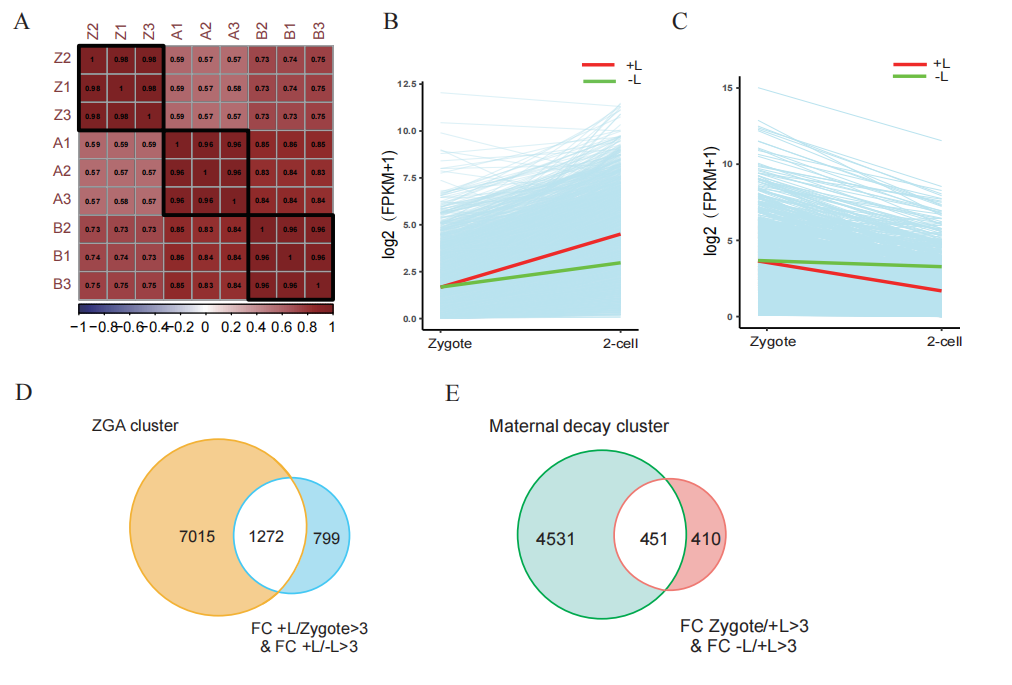


**Figure S1. The depletion of lactate leads to the failure of ZGA**

1. The correlation coefficient among the duplicates for zygotes, the +L group, and the -L group.
2. Expression patterns of mouse zygotic genomes during the zygote-2cell transition derived from the +L and -L groups. Each light blue line represents the expression levels of an individual gene, while the red and green lines depict the median expression levels of the +L and -L groups, respectively.
3. Degradation patterns of mouse maternal transcripts during the zygote-2cell transition derived from +L group and -L group. Each light blue line represents the expression levels of an individual gene, and the red, green lines represent the median expression levels of +L group and -L group, respectively.
4. Venn diagrams showing the overlap between ZGA genes published by Ken-ichiro Abe et al. ^30^ and transcripts meeting the criteria (fold change [+L/zygote] > 3 & fold change [+L/-L] in late 2-cell > 3).
5. Venn diagrams showing the overlap between maternal decay genes published by Ken-ichiro Abe et al. ^30^ and transcripts meeting the criteria (fold change [zygote/+L] > 3 & fold change [-L/+L] in late 2-cell > 3).


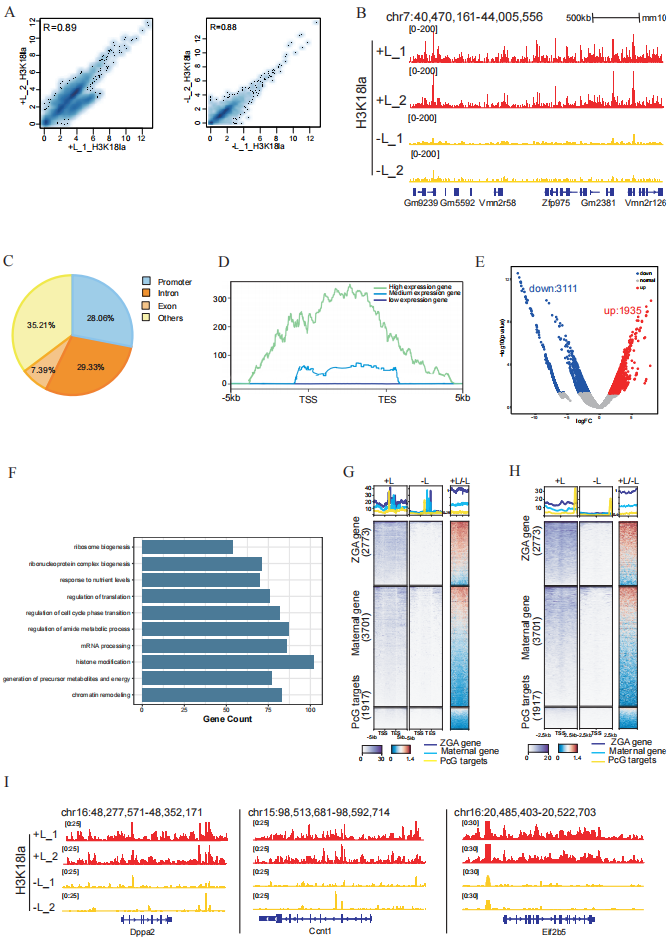


**Figure S2. The histone lactylation is responsible for the ZGA failure.**

1. Scatterplot correlation depicting CUT & Tag replicates (rep1 and rep2) in two groups, with and without sodium lactate.
2. A snapshot from the genome browser displaying the enrichment of H3K18la in the replicates of the two groups, with and without sodium lactate.
3. A pie chart illustrating the distribution of H3K18la signals in the genome.
4. Metaplot of H3K18la signals (Z-score normalized), presenting the average H3K18la enrichment along gene bodies and 5 kilobases (kb) upstream/ downstream of gene bodies categorized by high, mid, and low expression level in 2-cell embryos sorted by RPKM.
5. A volcano diagram displaying differential genes corresponding to the signals detected by the H3K18la CUT & Tag in the +L and -L groups.
6. Gene Ontology (GO) enrichment analysis for the down regulated genes in (E).
7. Heatmap illustrating the enrichment of H3K18la signals in the gene bodies of maternal decay genes, Polycomb Group (PcG) genes, and Zygotic Genome Activation (ZGA) genes between the +L and -L groups. Refer to Zhang Yi et al. for the gene list^29^. Average plots demonstrate the enrichment of H3K18la signals in the +L compared with -L group.
8. Heatmap showing the enrichment of H3K18la signals in the promoters of maternal decay genes, PcG genes, and ZGA genes between the +L and -L groups. Refer to Zhang Yi et al. for the gene list^29^. Average plots exhibit the enrichment of H3K18la signals in the +L compared with -L group.
9. Representative IGV snapshots illustrating the enrichment of H3K18la signal in both +L and -L groups.


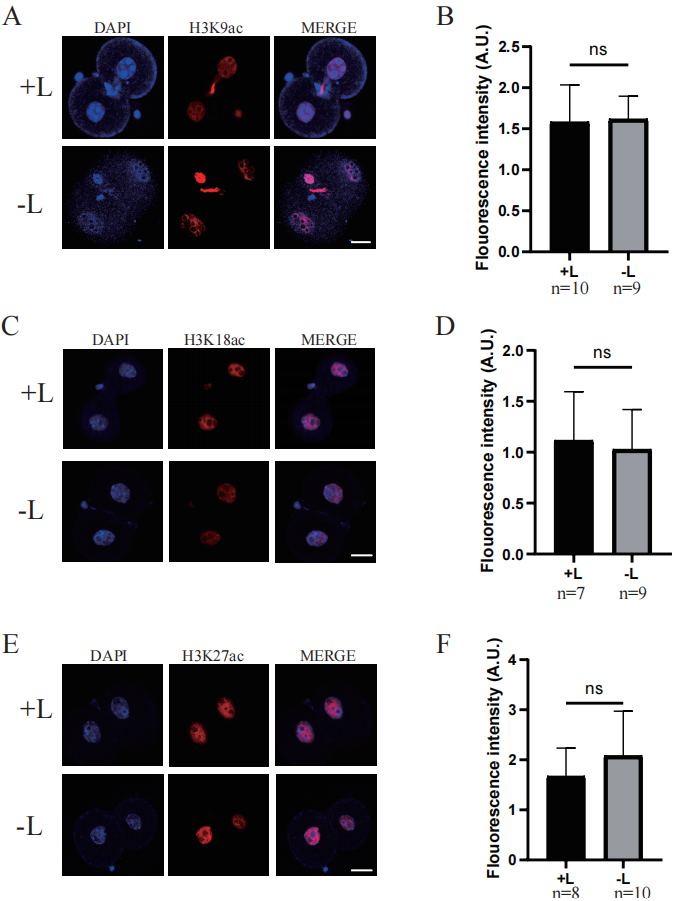


**Figure S3. There were no significant differences in histone acetylation modifications at the three sites in 2-cell embryos between the sodium lactate-treated and untreated groups.**

1. Images of embryos immunostained with antibodies against H3K9ac in the +L and -L groups. Scale bar: 20μm.
2. Bar plot showing the H3K9ac fluorescent intensity of 2-cell embryos derived from the +L and -L groups. ns: no significant.
3. Images of embryos immunostained with antibodies against H3K18ac in the +L and -L groups. Scale bar: 20μm.
4. Bar plot showing the H3K18ac fluorescent intensity of 2-cell embryos derived from the +L and -L group. ns: no significant.
5. Images of embryos immunostained with antibodies against H3K27ac in the +L and -L groups. Scale bar: 20μm.
6. Bar plot showing the H3K27ac fluorescent intensity of 2-cell embryos derived from the +L and -L group. ns: no significant.


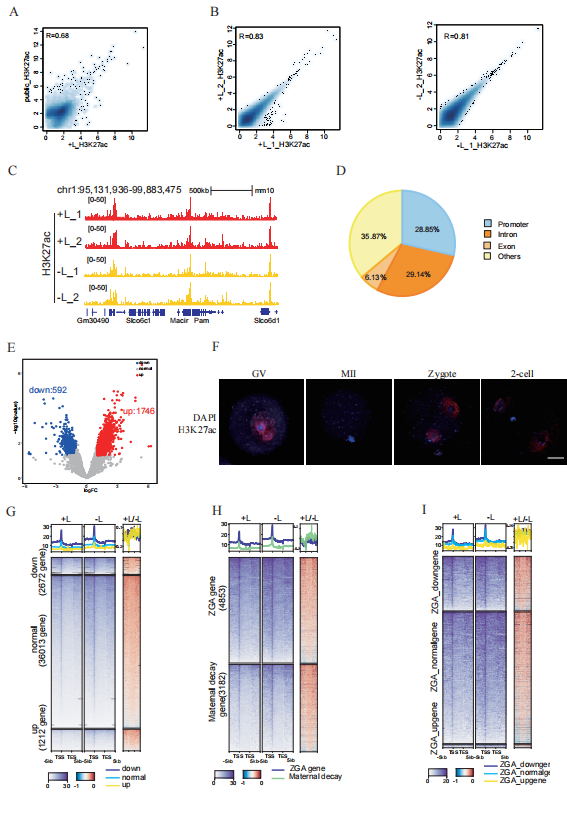


**Figure S4. H3K27ac does not responsible to ZGA failure due to lactate deficiency**

1. Scatterplot correlation of CUT & Tag in the +L group with publicly available data on 2-cell embryos ^29^.
2. Scatterplot correlation of CUT & Tag replicates (rep1 and rep2) in the two groups, with and without sodium lactate.
3. A snapshot from the genome browser displaying the enrichment of H3K27ac in the replicates of the two groups, with and without sodium lactate.
4. A pie chart illustrating the distribution of H3K227ac signals in the genome.
5. A volcano diagram displaying the differential genes corresponding to the signals detected by the H3K27ac CUT & Tag in the +L and -L groups.
6. Immunostaining of H3K27ac in mouse germinal vesicle (GV) oocytes, metaphase II (MII) oocytes, zygotes and late 2-cell embryos. Scale bar: 20 μm.
7. Heatmap showing the enrichment of H3K27ac signals in DEG gene bodies between the +L group and -L group. "Down," "normal," and "up" denote genes that are down-regulated, exhibit no significant difference, and are up-regulated, respectively, in the -L compared to +L. Average plots depict the enrichment of H3K27ac signals in the +L compared with -L group.
8. Heatmap showing the enrichment of H3K27ac signals in maternal decay genes (fold change [zygote/+L] > 3) and ZGA genes (fold change [+L/zygote] > 3) gene bodies between the +L group and -L group. Average plots demonstrate the enrichment of H3K27ac signals in the +L compared with -L group.
9. Heatmap showing the enrichment of H3K27ac signals in DEGs belonging to ZGA genes (fold change [+L/zygote] > 3) gene bodies between the +L group and -L group. Average plots show the enrichment of H3K27ac signals in the +L compared with -L group.


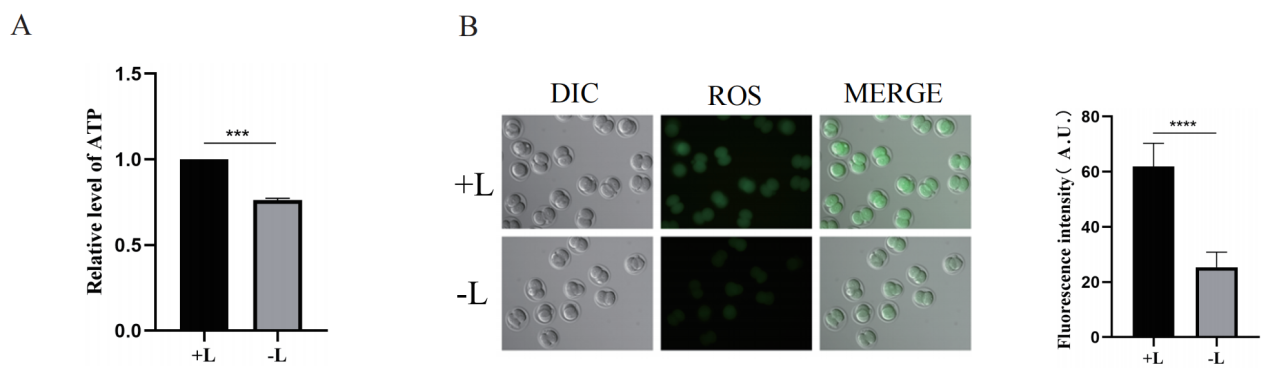


**Figure S5. Lactate deficiency may affect energy supply in early embryos**

1. Relative ATP levels in late 2-cell stage embryos for both +L and -L conditions.
2. Reactive Oxygen Species (ROS) levels in late 2-cell stage embryos for both +L and -L conditions. The right panel provides quantification of immunostaining relative signal intensities.

**Supplementary Information**

**Table S1.** The development rate of embryos cultured in medium by switching between +L and -L.

|  | Experiments | Embryos | 4-cell | Blastocyst |
| --- | --- | --- | --- | --- |
|  | （n） | (n) | （%） | （%） |
| -L/+L/-L | 3 | 87 | 45.9±3.5 | 35.6±2.1 |
| +L/-L/+L | 3 | 89 | 8.7±4.5*** | 3.3±3.3*** |

Zygotes cultured in –L (until 24hrs) and then shifted to +L (from 24 to 54hrs) and then shifted back to –L (after 54hrs) is called -L/+L/-L medium for simplicity; Zygotes cultured in +L (until 24hrs) and then shifted to -L (from 24 to 54hrs) and then shifted back to +L (after 54hrs) is called +L/-L/+L medium for short. Two-tailed Student’s t-test was used for the statistical analysis. ***P<0.001.

**Table S2.** Primers used for qPCR

| Gene | GeneBank No. (Ensemble ID) | Primer Pairs |
| --- | --- | --- |
| *Ccnb1* | NM 172301.3 | F: GCCTCACAAAGCACATGACTG  R: GTACAGTTCAGCTGTGCCA |
| *Cdk1* | NM 007659.4 | F: ACTCGGCCTCTAAGCTCCT  R: AGGTTACGACGGACCCTCTC |
| *Cdc25c* | NM 009860.3 | F: TGCCTGACGTCTATAGCCCC  R: TGCAGGTGGGATAGGTCCTG |
| *Wee1b* | NM 001356375.1 | F: CCGCACACTCCCAAGAGTT  R: TGGGGAGTTTGCCGTGTATC |
| *Dppa2* | NM 028615.1 | F: CACTCTTCGGGAGTGGTGTC  R: TTCTGGCCTCCCGAGATGTA |
| *Dppa4* | NM 028610.2 | F: AAGGCTAAAGCAACGGGGAA  R: GGTTCACTGGGGGCAGATAC |
| *Dux* | NM 001081954.1 | F: GGCCCTGCTATCAACTTTCAAGA  R: TTCTGAAACCACACGCGGA |
| *Sirt1* | NM 001159589.2 | F: CGGCTACCGAGGTCCATATAC  R: ACAATCTGCCACAGCGTCAT |
| *Ddx21* | NM 019553.2 | F: AACTTGCCCTCATTGGGTGT  R: CTTAATCGCCAGGTGCTCCA |
| *Hprt* | NM 013556.2 | F: GGCTTCCTCCTCAGACCGCTTT  R: CACTTTTTCCAAATCCTCGGCATAA |
